# Comparative genomics using *Fugu *reveals insights into regulatory subfunctionalization

**DOI:** 10.1186/gb-2007-8-4-r53

**Published:** 2007-04-11

**Authors:** Adam Woolfe, Greg Elgar

**Affiliations:** 1 School of Biological Sciences, Queen Mary, University of London, Mile End Road, London E1 4NS, UK; 2 Genomic Functional Analysis Section, National Human Genome Research Institute, National Institutes of Health, Rockville, MD 20870, USA

## Abstract

**Background:**

A major mechanism for the preservation of gene duplicates in the genome is thought to be mediated via loss or modification of *cis*-regulatory subfunctions between paralogs following duplication (a process known as regulatory subfunctionalization). Despite a number of gene expression studies that support this mechanism, no comprehensive analysis of regulatory subfunctionalization has been undertaken at the level of the distal *cis*-regulatory modules involved. We have exploited fish-mammal genomic alignments to identify and compare more than 800 conserved non-coding elements (CNEs) that associate with genes that have undergone fish-specific duplication and retention.

**Results:**

Using the abundance of duplicated genes within the *Fugu *genome, we selected seven pairs of teleost-specific paralogs involved in early vertebrate development, each containing clusters of CNEs in their vicinity. CNEs present around each *Fugu *duplicated gene were identified using multiple alignments of orthologous regions between single-copy mammalian orthologs (representing the ancestral locus) and each fish duplicated region in turn. Comparative analysis reveals a pattern of element retention and loss between paralogs indicative of subfunctionalization, the extent of which differs between duplicate pairs. In addition to complete loss of specific regulatory elements, a number of CNEs have been retained in both regions but may be responsible for more subtle levels of subfunctionalization through sequence divergence.

**Conclusion:**

Comparative analysis of conserved elements between duplicated genes provides a powerful approach for studying regulatory subfunctionalization at the level of the regulatory elements involved.

## Abstract 2

Fish-mammal genomic alignments were used to compare over 800 conserved non-coding elements that associate with genes that have undergone fish-specific duplication and retention, revealing a pattern of element retention and loss between paralogs indicative of subfunctionalization.

## Background

Gene duplication is thought to be a major driving force in evolutionary innovation by providing material from which novel gene functions and expression patterns may arise. Duplicated genes have been shown to be present in all eukaryotic genomes currently sequenced [1] and are thought to arise by tandem, chromosomal or whole genome duplication events. Unless the duplication event is immediately advantageous (for example, by gene dosage increasing evolutionary fitness), the gene pair will exhibit functional redundancy, allowing one of the pair to accumulate mutations without affecting key functions. Because deleterious mutations are thought to occur much more commonly than neutral or advantageous ones, the classic model for the evolutionary fate of duplicated genes [2,3] predicts the degeneration of one of the copies to a pseudogene as the most likely outcome (a process known as non-functionalization). Less commonly, a mutation will be advantageous, allowing one of the gene duplicates to evolve a new function (a process known as neo-functionalization). Therefore, the classic model predicts that these two competing outcomes will result in the elimination of most duplicated genes. However, several studies suggest that the proportion of duplicated genes retained in vertebrate genomes is much higher than is predicted by this model [4-6]. This has led to the suggestion of an alternative model whereby complementary degenerative mutations in independent subfunctions of each gene copy permits their preservation in the genome, as both copies of the gene are now required to recapitulate the full range of functions present in the single ancestral gene. This was formalized in the Duplication-Degeneration-Complementation (DDC) model [7] in a process referred to as subfunctionalization.

The key novelty of the DDC model is that, rather than attributing different expression patterns of duplicated genes to the acquisition of novel functions, they are attributed to a partial (complementary) loss of function in each duplicate. In combination they retain the complete function of the pleiotropic original gene, but neither of them alone is sufficient to provide full functionality. For this model to be viable, the subfunctions of the gene are required to be independent so that mutations in one subfunction will not affect the other. The modular nature of many eukaryotic protein-coding sequences as well as *cis*-regulatory modules (CRMs), such as enhancers or silencers [8], means both can act as subfunctions or components of subfunctions of the gene in subfunctionalization. CRMs are *cis*-acting DNA sequences, up to several hundred bases in length, thought to be composed of clustered combinatorial binding sites for large numbers of transcription factors that together actuate a regulatory response for one or more genes [9]. The larger number of independently mutable units represented by CRMs, the small size and rapid turnover of transcription factor binding sites, as well as observations that, for many gene duplicates, changes that occur between paralogs are due to changes in expression rather than protein function has led a number of researchers to emphasize that important evolutionary changes might occur primarily at the level of gene regulation [10,11]. Consequently, subfunctionalization is thought most likely to occur by complementary degenerative mutations within regulatory elements.

Teleost fish provide an excellent system to study the DDC model in vertebrates due to the presence of extra gene duplicates that derive from a whole genome duplication event early in the evolution of ray-finned fishes 300-350 million years ago [12-17] This provides the opportunity for comparative analyses of gene duplicates in fish against a single ortholog in tetrapod lineages such as mammals. In particular, for analyses involving important developmentally associated genes, these 'single copies' represent as close as possible the ancestral gene from which the fish duplicates descended, since such genes are often highly conserved in sequence and function throughout vertebrates. We therefore refer to fish-specific duplicate genes as 'co-orthologs' (a term previously used in [18]) as each copy is co-orthologous to the single homolog in tetrapods.

A number of studies on fish duplicated genes have identified cases of subfunctionalization at both the regulatory and protein level. For instance, analysis of the *synapsin-Timp *genes in the pufferfish *Fugu rubripes *identified a case of protein subfunctionalization where two isoforms of the *SYN *gene expressed in human are expressed as two separate genes in *Fugu *[19]. A number of functional studies on the shared and divergent expression patterns of developmental co-orthologs in fish have also been carried out, for example, *eng2 *[20], *sox9 *[18] and *runx2 *[21]. In each case, partitioning of ancestral expression domains for each co-ortholog compared to the single (ancestral representative) gene in mammals was observed via gene expression studies, supporting a process of regulatory subfunctionalization along the lines of the DDC model. Work on identifying the regulatory elements involved has so far been limited to those responsible for divergent expression within the well-studied Hox genes. Santini *et al*. [22], through comparison to the single tetrapod Hox cluster, identified a number of conserved elements in fish-specific Hox clusters. These appeared to be partitioned between clusters, suggesting they may be responsible for their divergent expression. In addition, the zebrafish *hoxb1a *and *hoxb1b *genes, co-orthologs of the *HOXB1 *gene in mammals and birds, were found to exhibit complementary degeneration of two *cis*-regulatory elements identified upstream and downstream of the gene, consistent with the DDC model [23]. Similarly, Postlethwait *et al*. [24] carried out a comparative genomic analysis of the regions surrounding two zebrafish co-orthologs, *eng2a *and *eng2b*, against the single human ortholog *EN2 *and found one conserved non-coding element partitioned in each copy, together with a number of elements conserved in both. Both co-orthologs have overlapping expression in the midbrain-hindbrain border and jaw muscles, but *eng2a *is expressed in the somites and *eng2b *is expressed in the anterior hindbrain (both of which are expression domains found in the single mammalian ortholog). Hence, according to the DDC model, they hypothesized that sequences conserved in both co-orthologs represent regulatory elements responsible for overlapping expression domains, whilst conserved sequences specific to each gene are candidates for regulatory elements that drive expression to domains present in the single mammalian ortholog but now partitioned between co-orthologs. Despite these isolated examples, evidence for the DDC model, by way of identifying the regulatory elements responsible, remains limited.

Comparison of non-coding genomic sequence across extreme evolutionary distances such as that between fish and mammals to identify regions that remain conserved has proved powerful in identifying sequences likely to be vertebrate-specific distal CRMs (see [25] for a review). *Fugu*-mammal conserved non-coding elements (CNEs), identified genome-wide, cluster almost exclusively in the vicinity of genes implicated in transcriptional regulation and early development (termed *trans-dev *genes) with little or no conservation in non-coding sequence outside of these regions; a finding confirmed by a number of recent studies [25-31]. Furthermore, a majority of those CNEs tested *in vivo *drive expression of a reporter gene in a temporal and spatial specific manner that often overlaps the endogenous expression pattern of the nearby *trans-dev *gene, confirming this association and their likely role as critical CRMs for these genes [26,29,32-36]. The tight association of CNEs with *trans-dev *genes is likely the result of the fundamental nature of developmental gene regulatory networks involved in correct spatial-temporal patterning of the vertebrate body plan [26,37].

*Fugu*-mammal CNEs, enriched for putative CRMs, therefore provide an excellent class of sequences through which to test the DDC model further. In addition, a study has found that at least 6.6% of the *Fugu *genome is represented by fish-specific duplicate genes [15], making *Fugu *an attractive genome in which to identify and analyze regulatory elements involved in subfunctionalization of fish co-orthologs. Transcription factors and genes involved in development and cellular differentiation appear to be overrepresented within duplicated genes in fish genomes [38], improving the chances of identifying suitable candidates. Here, by taking an approach similar to Postlethwait *et al*. [24], we carried out alignments of genomic sequence around seven pairs of *Fugu *developmental co-orthologs against a number of single mammalian orthologous regions in order to investigate whether differential presence of conserved elements between co-orthologs is consistent with the DDC model of regulatory subfunctionalization.

## Results

### Identification of co-orthologs in the *Fugu *genome

Studies into fish-specific duplicated genes have identified a number of examples in the *Fugu *genome (for example, [15,39]). As with most genes in general, few of these *Fugu *specific duplicates have CNEs in their vicinity. Suitable gene candidates for study of CNE evolution between teleost-specific gene paralogs were initially identified using 2,330 CNEs derived from a whole-genome comparison of the non-coding portions of the human and *Fugu *genome [29]. CNE clusters that mapped to the vicinity of a single human genomic region but were derived from two non-contiguous *Fugu *scaffolds were considered further. We selected seven genomic regions in human that fitted this criterion, each containing clusters of CNEs in the vicinity of a single gene implicated in developmental regulation: *BCL11A *(transcription factor B-cell lymphoma/leukemia 11A), *EBF1 *(early B-cell factor 1), *FIGN *(fidgetin), *PAX2 *(paired box transcription factor Pax2), *SOX1 *(HMG box transcription factor Sox1), *UNC4.1 *(homeobox gene Unc4.1) and *ZNF503 *(zinc-finger gene Znf503). Some of these genes have relatively well characterized roles in early development, such as *PAX2 *(which plays critical roles in eye, ear, central nervous system and urogenital tract development [40-42], *SOX1 *(involved in neural and lens development [43,44], *BCL11A *(thought to play important roles in leukaemogenesis and haematopoiesis [45]) and *EBF1 *(important for B-cell, neuronal and adipocyte development [46,47]. *FIGN*, *UNC4.1 *and *ZNF503 *are less well characterized, although studies of their orthologs in mouse or rat indicate important roles in retinal, skeletal and neuronal development [48-51].

**Figure 1:**
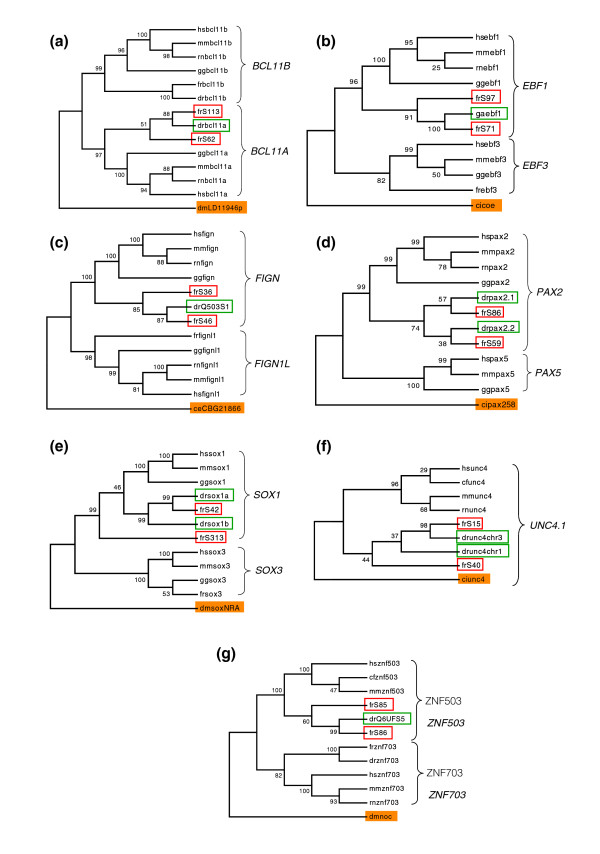
Phylogenies of seven *Fugu *co-orthologs. *Fugu *(fr) co-ortholog protein sequences are highlighted by red boxes and named according to scaffold number they were located on (for example, frS86 = scaffold_86). Zebrafish (dr) or stickleback (ga) sequences are highlighted by green boxes and uncharacterized proteins named after the SwissProt ID or the chromosome they are located on. Bootstrap values are indicated at each node. Other tetrapod sequences included: human (hs), mouse (mm), rat (rn), dog (cf) and chicken (gg). Invertebrate outgroups are shaded orange and contain sequences from the following species: *Ciona intestinalis *(ci), *Drosophila melanogaster *(dm) and *Caenhoribditis elegans *(ce). Trees: **(a) ***BCL11A *using the closest paralog *BCL11B *as a comparator. **(b) ***EBF1 *using the closest paralog *EBF3 *as a comparator. **(c) ***FIGN *using the closest paralog *FIGN1L *as a comparator. **(d) ***PAX2 *using one of its two closest paralogs *PAX5 *as a comparator. **(e) ***SOX1 *using its closest paralog *SOX3 *as a comparator. **(f) ***UNC4.1 *has no known closely related paralogs. **(g) ***ZNF503 *using its closest paralog *ZNF703 *as a comparator.

For each CNE cluster region in the human genome, we identified homologs to the human *trans-dev *protein on each *Fugu *scaffold, suggesting the presence of co-orthologous genes. To confirm this, we carried out a phylogeny of these protein sequences together with tetrapod orthologs and all available co-orthologs from the zebrafish genome. In addition, two outgroups utilizing the closest in-paralog as well as an invertebrate ortholog were included in each alignment to help resolve the phylogeny (Figure 1). In all cases where a close paralog could be identified, the *Fugu *co-ortholog candidates branch with strong bootstrap values with tetrapod orthologs of the target *trans-dev *gene, rather than the closest paralog, confirming these genes are true co-orthologs. Furthermore, for all phylogenies, the *Fugu *and zebrafish/medaka sequences branch together after the split with tetrapods, confirming they derive from a fish-specific duplication event. In only one out of three cases (*pax2*) where two co-orthologous proteins could also be identified in zebrafish does each *Fugu *copy branch directly with each zebrafish copy, indicating their proteins have followed similar evolutionary paths (Figure 1d). In contrast, the other two cases (*sox1 *and *unc4.1*) exhibit a different topology in that both zebrafish co-orthologs are more similar to one of the *Fugu *co-orthologs than the other (although weak bootstrap values for the fish *unc4.1 *may suggest alternative phylogenies). This is most likely due to species-specific asymmetrical rates of evolution seen between many genes in teleost fish [52], as well as elevated rates of evolution in duplicated genes in general, and pufferfish in particular [38], which may have obscured the true phylogenies in these cases. The given names of the *Fugu *co-orthologs used in this study (see Materials and methods for more details on nomenclature), their location in the *Fugu *genome and protein sequence accession codes can be found in Table 1.

### CNE distribution and changes in genomic environment around *Fugu *co-orthologs

**Figure 2:**
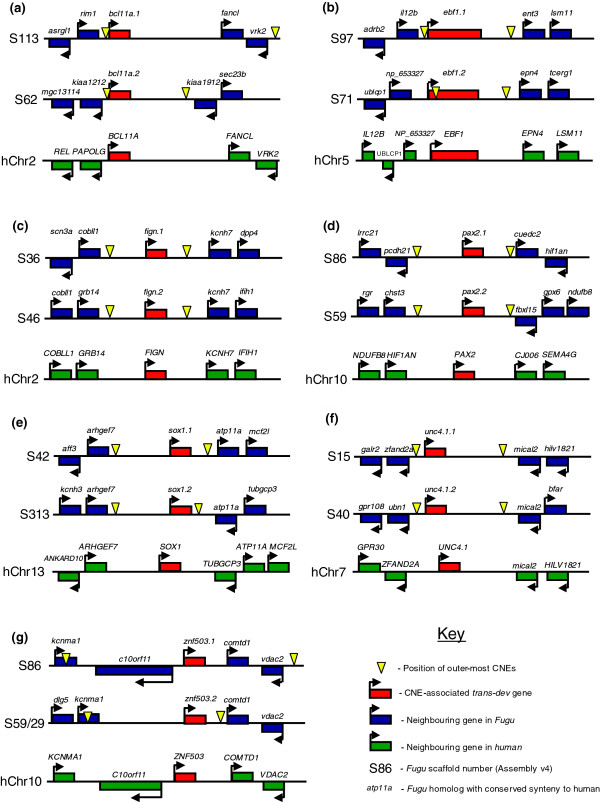
Genomic environment around *Fugu *co-orthologs in comparison to the human ortholog. Diagrammatic representation of the genomic environment around *Fugu *co-orthologs and human orthologs of: **(a) ***BCL11A*, **(b) ***EBF1*, **(c) ***FIGN*, **(d) ***PAX2*, **(e) ***SOX1*, **(f) ***UNC4.1 *and **(g) ***ZNF503*. For each gene, the top two lines represent the genic environment around each of the *Fugu *co-orthologs whilst the third line represents the genic environment around the human ortholog. Regions are not drawn to scale and are representative only. Human chromosome locations and *Fugu *scaffold IDs are stated to the left of each graphic. *Fugu *scaffold IDs can be cross-referenced for their exact location through Table 1. All annotation was retrieved from Ensembl *Fugu *(v36.4) and Human (v.36.35i). Only genes that are conserved in both *Fugu *and human are shown. Reference *trans-dev *genes are colored in red and are always orientated in 5'→3' orientation. Surrounding genes in *Fugu *are marked in blue and in human in green. The names of neighboring *Fugu *homologs that share conserved synteny with human (but not necessarily the same relative order or orientation) are highlighted in an orange box. Genes orientated in the same direction as the reference *trans-dev *gene are located above the line and those orientated in the opposite direction are below the line. Yellow triangles represent the positions of the furthest CNEs upstream and downstream in each genomic sequence and delineate the region in which CNEs were identified.

CNEs were independently identified within each *Fugu *co-orthologous region by carrying out a combination of multiple and pairwise alignment with the same orthologous sequence from human, mouse and rat (the entire dataset from this study can be accessed and queried through the web-based CONDOR database [53]). The regions in which CNEs were located for each co-ortholog together with surrounding gene environment can be seen in Figure 2.

All but one of the CNE regions in human are located in gene-poor regions termed 'gene deserts' that flank or surround the *trans-dev *gene and are characteristic of regions thought to contain large numbers of *cis*-regulatory elements [30]. These gene deserts appear to have been conserved to some degree in both *Fugu *copies (albeit in a highly compact form). For example, a large gene desert of approximately 2.2 Mb is located downstream of *BCL11A *up to the ubiquitin ligase gene *FANCL *in human, and similar (compacted) versions of this gene desert are present in both *Fugu *regions, although downstream of *bcl11a.2 *it is almost a quarter of the size compared to the same region in *bcl11a.1 *(98 kb versus 380 kb). In the majority of regions under study (five out of seven), CNEs extend purely within these large intergenic regions directly flanking or within the introns of the *trans-dev *gene. In those regions in which CNEs extend beyond or within the genes neighboring the *trans-dev *gene (that is, *bcl11a.1*, *znf503.1 *and *znf503.2*) the gene order and orientation between *Fugu *and human has remained largely conserved, spanning three to five genes, something that is relatively rare within the *Fugu *genome [54,55]. This may be due to functional constraints on these regions whereby it is necessary to maintain the CRM and associated gene in *cis *[34,56]. For the remaining co-orthologous regions the degree of synteny varies widely. For instance, neither *Fugu pax2 *region has conserved gene order with the human genome. Two orthologs of *NDUFB8 *and *HIF1AN *(upstream of human *PAX2*) are partitioned and rearranged so that *hif1an *is downstream of *pax2.1 *and *ndufb8 *is downstream of *pax2.2 *(Figure 2).

The preservation of 98.5% of the CNEs (796/811) as well as both *trans-dev *genes in the same orientation and order along the sequence between human and *Fugu*, in contrast to the rearrangement of surrounding genes, confirms the likelihood that the CNEs and *trans-dev *genes identified are associated with each other.

### Pattern of CNE retention/partitioning between co-orthologs

**Figure 3:**
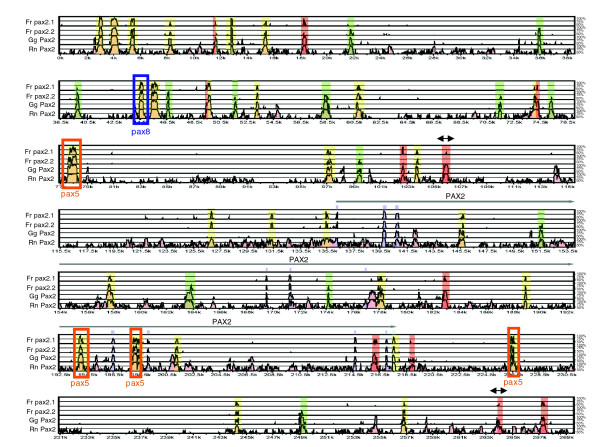
VISTA plot of an MLAGAN alignment of orthologous regions surrounding two *pax2 *co-orthologs in *Fugu *(Fr) and *Pax2 *in chicken (Gg), rat (Rn) and human. The baseline is 268 kb of human sequence. Conservation between human and each sequence is shown as a peak. Peaks that represent conservation in a non-coding region of at least 65% over 40 bp are shaded pink with coding exons shaded purple and peaks located within untranslated regions shaded light-blue. All CNEs conserved in at least one of the *Fugu *co-orthologs are color-coded. CNEs in both *Fugu *co-orthologs that overlap the same region in human are shaded yellow while CNEs that are 'distinct' (or conserved solely) in *pax2.1 *are shaded red and CNEs distinct to *pax2.2 *are shaded green. Peaks marked with a double-headed arrow are conserved in *Fugu *in the opposite orientation (and therefore do not show up in the VISTA plot). A number of the CNEs around *PAX2 *are also duplicated CNEs (dCNEs) that are located elsewhere in the genome in the vicinity of *PAX2 *paralogs. CNEs marked with an orange box have another dCNE family member in the vicinity of *PAX5 *and the CNE marked with a blue box has a dCNE family member conserved upstream of *PAX8*.

The DDC model for the retention of gene duplicates over evolution states that following duplication, genes undergo complementary degenerative loss of subfunctions or, on the regulatory level, expression domains. Based on the assumption that CNEs represent putative autonomous CRMs that control gene expression to one or more specific expression domains, we would predict that this process of regulatory subfunctionalization would involve the degeneration or loss of these elements between gene duplicates so that the ancestral CRMs were to some degree partitioned between the two genes. We identified 811 CNEs in total for all 14 regions in *Fugu *with lengths ranging from 30-562 bp (mean = 117 bp, median = 85 bp) and human-*Fugu *percent identities ranging from 60-94% (mean = 74%). CNEs from each co-ortholog were defined as 'overlapping' if there was conservation between them to at least part of the same single sequence in human. CNEs that were conserved between human and only one *Fugu *co-ortholog with no significant overlap to CNEs in the counterpart co-ortholog were defined as 'distinct'. Figure 3 illustrates the definition of overlapping and distinct CNEs identified in a multiple alignment between *Fugu *regions around *pax2.1 *and *pax2.2*, against the reference human *PAX2 *region.

**Figure 4:**
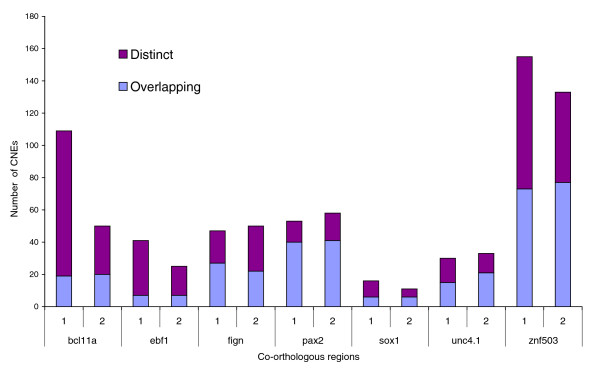
Proportion of CNEs around each *Fugu *co-ortholog that overlap or are distinct to sequences in mammals compared to CNEs identified in its counterpart co-ortholog. Each bar represents the total number of CNEs identified around each co-ortholog with a proportion of that total colored as overlapping (light purple) or distinct (maroon) CNEs.

Similar to other *trans-dev *gene regions identified previously (for example, [26]), the co-orthologs under study have highly variable numbers of CNEs conserved in their vicinity, ranging from 11 CNEs in *sox1.2 *to 156 in *znf503.1 *(Figure 4). Comparison of the overall number of CNEs conserved between co-orthologous copies revealed three sets, *bcl11a*.*1/2*, *ebf1.1/2 *and *znf503.1/.2*, that have notably different overall numbers of CNEs located in their vicinity, indicating a large-scale loss of elements in one co-ortholog compared to its counterpart since duplication (Figure 4). In the cases of *bcl11a.1/2 *and *znf503.1/2*, this large-scale asymmetrical loss of elements in one co-ortholog copy correlates to a large decrease in genomic sequence within the same region (Additional data file 2). Many of the co-orthologs have also undergone substantial partitioning of elements, as indicated by the large proportion of the identified CNEs classified as 'distinct' in each co-ortholog. For example, *fign.1 *and *fign.2 *have a similar number of CNEs in their vicinity (47 and 50, respectively) but 42% and 56% of these CNEs, respectively, are distinct to each co-ortholog. The extent of distinct CNEs as a proportion of total CNEs differs significantly between sets of co-orthologs, ranging from 24.5% (13/53) in *pax2.1 *to 83% (34/41) in *ebf1.1 *(Figure 4). For co-orthologs of *BCL11A *and *EBF1 *the majority of CNEs in both genes are distinct. Only in co-orthologs of *PAX2 *are the majority of CNEs in both genes found to be overlapping (Figures 3 and 4), suggesting a high level of retention of regulatory domains in both genes since duplication. In the majority of gene pairs, namely co-orthologs of *FIGN*, *SOX1*, *UNC4.1 *and *ZNF503*, one copy has the majority of its CNEs as distinct while the other has a majority of its CNEs overlapping with that of its counterpart co-ortholog, suggesting an asymmetrical rate of element partition.

The accuracy of these results depends heavily on ensuring that the loss of elements in one co-ortholog is the result of subfunctionalization rather than lack of sequence coverage in the genomic sequence. The proportion of 'N's (sections of unfinished sequence) within each *Fugu *genomic sequence can be seen in Table 1. We found that only one of the gene regions, *sox1.2*, contains a significant proportion of unfinished sequence (8.9%), suggesting some of the CNEs defined as 'distinct' in *sox1.1 *may have overlapping counterparts in *sox1.2*. However, closer examination of the positioning of the unfinished sequence reveals that the vast majority occurs in a region easily defined by two flanking overlapping CNEs that contains just a single distinct CNE in its counterpart co-ortholog. The region in *sox1.2 *potentially containing counterparts to most of the distinct CNEs in *sox1.1 *contains less than 3% unfinished sequence, suggesting most, if not all, of these distinct CNEs are defined correctly. Without 100% finished sequence in all cases it is, of course, possible that a small proportion of the CNEs identified as distinct in these co-orthologs may have an overlapping counterpart within unfinished sequence, but given the high levels of finished sequence in most of the gene regions, this is unlikely to account for a significant number.

### Evolution of overlapping CNEs since duplication

Overlapping CNEs comprise a large proportion and, in some cases, the majority of CNEs identified around many of the gene pairs and have, therefore, remained to some extent under positive selection in both co-orthologs. The distribution of lengths and percent identities for 381 overlapping CNEs versus 430 distinct CNEs is significantly different for both lengths (*p *< 1 × 10^-16^) and percent identities (*p *= 1.1^-8^). Overlapping CNEs have significantly higher average lengths (mean = 149.6 bp, median = 116.1 bp) than distinct CNEs (mean = 87.6 bp, median = 62 bp) as well as slightly higher percent-identities (mean = 75.2% and median = 75% for overlapping versus mean = 72.4% and median = 71.7% for distinct). Only 4 of the distinct CNEs overlap to some degree but by less than the arbitrary 20 bp cut-off required for CNEs to be defined as overlapping. Removing these leaves the mean lengths and percent-identities virtually unchanged, confirming that the cut-off did not significantly bias the distribution of distinct elements towards smaller elements.

We studied two aspects to gauge evolutionary changes occurring in these elements since duplication: changes in element length and changes in substitution rate between overlapping CNEs in *Fugu*.

### CNE length

**Figure 5:**
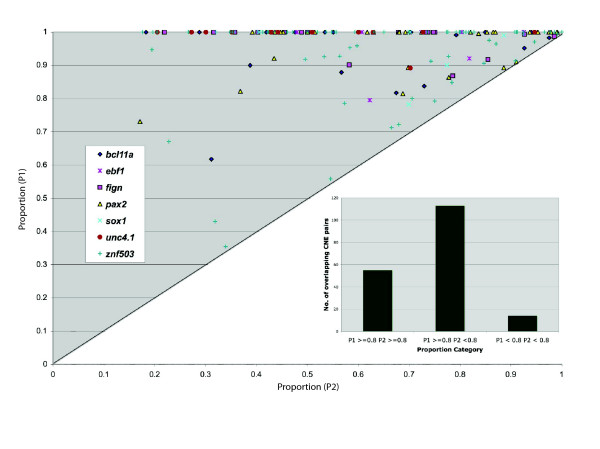
Proportion of each CNE sequence that overlaps the counterpart co-ortholog CNE. Main graph: for each overlapping pair of co-orthologous CNEs (involving just two sequences), the proportion of the full length of each CNE (P1-P2) made up by the overlap was calculated using the human sequence as the reference. The larger of the two proportions was always plotted as P1 to simplify analysis. Inset bar chart: summary of the number of overlapping CNE pairs falling into three main proportion categories: P1 ≥ 0.8, P2 ≥ 0.8 - pairs that overlapped over the majority of both elements, suggesting little evolution of element length since duplication; P1 ≥ 0.8, P2 < 0.8 - pairs that have undergone significant degeneration in element length in one of the copies compared to its counterpart; P1 < 0.8, P2 < 0.8 - pairs that have undergone a level of degeneration in element length in both copies at their edges.

**Figure 6:**
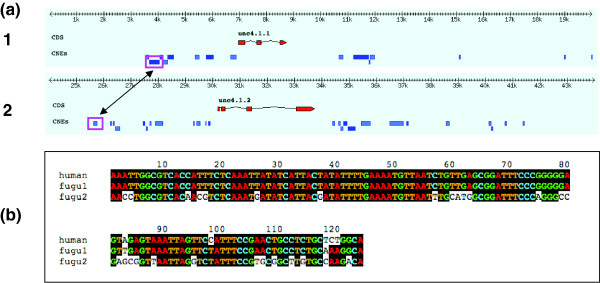
Significant change in element length and substitution rate in overlapping CNEs upstream of *unc4.1.1 *and *unc4.1.2*. **(a) **CNEs (filled blue boxes) were identified around each *Fugu *co-ortholog *unc4.1.1 *(A1, top) and *unc4.1.2 *(A2, bottom) (gene exons are shown in the coding sequence (CDS) track as filled red boxes). The scale at the top represents positions along the *Fugu *sequence used in the multiple alignment. Two CNEs, highlighted in pink boxes, one upstream of *Fugu unc4.1.1 *(CRCNEAC00031954 [53], referred to as CNE_A1) and one upstream of *unc4.1.2 *(CRCNEAC00032205 [53], referred to as CNE_A2) are conserved to part of the same sequence in human upstream of *UNC4.1*. The overlap region is 126 bp in length and encompasses all of the CNE_A2 but only 35% of CNE_A1 (which is 360 bp long), indicating a significant loss of element length in CNE_A2. **(b) **A relative rate test of the *Fugu *CNEs across the overlapping region using human as the outgroup reveals a highly significant number of independent substitutions (26) in CNE_A2 with no independent substitutions in CNE_A1 (*p *< 0.001). This suggests CNE_A1 is likely to have retained the ancestral function while CNE_A2 may have evolved to have a different function.

A total of 182 pairs of overlapping CNEs were identified across all co-ortholog pairs with a one-to-one relationship. The length of the overlap in the human sequence between co-orthologous CNEs ranged from 24-460 bp (mean = 107.5 bp ± 2.27 standard error of the mean). For each overlapping pair, we calculated the proportion of the overlapping sequence as a function of the full length *Fugu*-human conserved sequence in each co-ortholog. We found 62% of the pairs to have undergone significant degeneration in element length in one of the copies compared to its counterpart (Figures 5 and 6); 30% of pairs overlapped over the majority of both elements, suggesting little evolution of element length since duplication, and approximately 8% have undergone a significant level of degeneration in element length in both copies at their edges. These results suggest the process of subfunctionalization may also be occurring, at least in some of these cases, through the partial loss of function in both copies, allowing gene preservation through quantitative complementation (as suggested in [7]). It is also possible that sequence loss could causes changes in module function through the change in binding site combinations present. In genes such as *pax2.1 *and *pax2.2 *that have the majority of their CNEs overlapping in both genes, this presents an additional mechanism by which both copies may be preserved. In addition to overlapping CNEs that have undergone evolution at their edges, 29 overlapping CNEs have undergone evolution at the centre of the element, essentially creating a split element (that is, a CNE in one co-ortholog overlaps two or more CNEs from the other co-ortholog).

### CNE sequence evolution

Overlapping CNEs are conserved to the same human sequence across the length of the overlap. However, it is possible that elements have undergone differential evolution, with one element containing a significantly greater number of independent substitutions than the other, indicative of either subfunctionalization or neofunctionalization. To measure whether the sequence of one CNE has diverged faster than its counterpart, we used the Tajima relative rate test [57] with the human sequence as the outgroup (or ancestral) sequence. The Tajima relative rate test measures the significance in the difference of independent substitutions in each sequence relative to the outgroup sequence using a chi-squared statistic (see Additional file 3 for the results of relative rate tests for all overlapping CNEs). The percentages of overlapping CNEs that show a statistically significant difference in substitution rate in one copy over another range from 17% in *sox1 *to 26% in *znf503 *(Table 2). One of the most significant examples within this set was found in a pair of CNEs upstream of co-orthologs of *UNC4.1 *and can be seen in Figure 6. These results suggest that a substantial number of the elements appear to have undergone an asymmetrical rate of evolution since duplication, something we would expect under the DDC model. Alternatively, if these changes were positively selected it may indicate a process of neofunctionalization whereby co-orthologs have evolved novel regulatory patterns to that of the ancestral copy.

### A history of duplications: some co-orthologous CNEs were duplicated in ancient events at the origin of vertebrates

In addition to being involved in a teleost-specific duplication event, a number of the CNEs identified around the *trans-dev *genes in this study have been previously retained from ancient duplications thought to have occurred at the origin of vertebrates. While the majority of CNEs are single copy in the human genome, a recent study identified 124 families of CNEs genome-wide that have more than one copy across all available vertebrate genomes and are referred to as 'duplicated CNEs' (dCNEs) [29]. dCNEs are associated with nearby *trans-dev *paralogs and a number have been shown to act as enhancers that drive *in vivo *reporter-gene expression to similar domains [29]. The absence of these sequences in non-vertebrate chordate genomes and their association with paralogs that arose from whole-genome duplication events at the origin of vertebrates [58] places their origins sometime prior to this event more than 550 million years ago. The conservation of these elements over such extreme evolutionary distances suggests they play critical roles in the regulation of paralogs that have since undergone neofunctionalization. We found 30 non-redundant human CNEs (conserved to 52 co-orthologous CNEs in *Fugu*) to be dCNEs in the vicinity of one or more paralogs of the nearby *trans-dev *gene (Table 3). This further confirms the tight association of these CNEs with their nearby *trans-dev *genes as dCNEs resolve the CNE-gene association more clearly [59]. These dCNEs were identified in five of the seven co-orthologous regions with some dCNEs associated with more than one paralog (for example, *PAX2 *associated dCNEs located in the vicinity of *PAX5 *and *PAX8*; Table 3; Figure 3). 80% of the co-ortholog CNEs identified as dCNEs (42/52) are conserved in both co-ortholog regions in *Fugu*, a two-fold enrichment (*p *< 0.001) over the expected number given the overall proportions of overlapping and distinct elements in the CNE dataset.

## Discussion

Recent studies show there are a surprisingly large number of duplicated genes present in the genomes of all organisms that cannot be accounted for by the classic models of nonfunctionalization and neofunctionalization. The presence of large numbers of duplicated genes within the genomes of teleost fish, now widely presumed to have undergone a whole genome duplication event around 300-350 million years ago, provide an excellent opportunity for comparative studies to test the DDC model. Prior to the availability of large-scale genomic sequences, the ability to study regulatory subfunctionalization through identifying the regulatory elements responsible was limited due to a lack of appropriate identification strategies. The discovery of thousands of CNEs conserved across the vertebrate lineage, highly enriched for sequences likely to be distal *cis*-regulatory modules, allowed us to develop a strategy to begin to uncover this. We identified potential gene candidates that contain both CNEs in their vicinity and are likely to derive from fish-specific duplication events using data from the initial whole genome comparison of the *Fugu *and human genomes. CNEs that cluster in the same location in human but derive from two separate locations in the *Fugu *genome strongly indicate the presence of co-orthologous regions. We selected seven clusters of CNEs in the human genome, each in the vicinity of a single *trans-dev *gene that fulfilled these criteria. For each of these genes, we recreated a phylogeny using protein sequences identified in each *Fugu *region, confirming the genes are both orthologs (co-orthologs) of the single mammalian copy, and all topologies confirmed the genes derive from a duplication event following the split between ray-finned and lobe-finned fishes. This relationship was further confirmed by comparison of the genic environment around the *trans-dev *gene in both *Fugu *regions to that of the single region in human. Conserved gene order extends, in many cases, to one or more genes upstream and/or downstream of each co-ortholog, indicating a shared ancestral origin, although in several instances the neighboring genes have been partitioned between the co-orthologous regions, undergone rearrangement or have been lost.

The process of subfunctionalization, as described in the DDC model, is defined as the fixation of complementary loss of subfunctions that result in the joint preservation of duplicate loci [7]. For regulatory subfunctionalization, a subfunction may represent expression of a gene in a specific tissue, cell lineage or temporal stage. For genes with complex regulation these subfunctions are controlled by one or a combination of *cis*-regulatory modules. A proportion of these can be predicted through the comparative genomic approaches outlined in this study and for the purposes of this discussion are assumed to be represented by CNEs. Under the DDC model, subfunctionalization is thought to occur by two different routes: qualitative and quantitative [7]. Under qualitative subfunctionalization one duplicate copy undergoes one or more complete loss-of-subfunction mutations with the second copy subsequently acquiring null-mutations for a different set of subfunctions. Thus, each copy is required to recapitulate the full set of ancestral subfunctions. Under this route, a conserved element representing an independent *cis*-regulatory module that undergoes a null-mutation in one of the gene copies will no longer be under selective constraint, and will be 'lost' (that is, will not be detectable by sequence conservation) through the accumulation of degenerative mutations over evolution. This process should, therefore, be evident by the effective partitioning of conserved elements between co-orthologs. In contrast, quantitative subfunctionalization is more subtle and results from the fixation of reduction-of-expression mutations in both duplicates [7]. Here, both regulatory modules must be maintained in the genome once the summed activity for a particular subfunction in both copies has been reduced to the original level in the single ancestral gene.

By comparing each *Fugu *co-ortholog with its single orthologous region in mammals, we attempted to identify those 'ancestral' *cis*-regulatory modules present in the mammalian copy that are retained in only one of the *Fugu *copies and those that have to some extent been retained in both copies. This approach is particularly appropriate for early developmental regulators for which function and regulation are likely to be highly constrained across all vertebrates and the mammalian gene represents as close as possible the ancestral pre-duplication state. The probability of the preservation of gene duplicates through subfunctionalization is also assumed to be higher in genes with complex regulation that contain large numbers of independently mutable CRMs [60], a view reinforced by the overrepresentation of genes involved in development and cellular differentiation found in fish-specific gene duplicates [38]. As with *trans-dev *genes across the genome, overall numbers of CNEs between gene pairs differs substantially and is likely to reflect differences in regulatory complexity or the extent to which regulation in these genes has been conserved across vertebrate evolution. All seven pairs of co-orthologs contained a number of CNEs that have been partitioned into each co-ortholog along the lines of the qualitative subfunctionalization model. The extent to which CNEs have been partitioned between co-orthologs, however, varies widely. For some co-ortholog pairs, such as *bcl11a *and *ebf1*, the majority of CNEs appear to be completely partitioned between the two genes, whilst for other pairs, such as *pax2*, only a relatively small proportion is. In the DDC model, following initial subfunctionalization, the process of null-mutation fixation in persisting redundant subfunctions is thought to be random, leaving a roughly equal number of subfunctions in each gene copy. Although this appears to be true for the *fign*, *pax2 and unc4.1 *co-orthologs, partitioning in *bcl11a.1/.2 *and *ebf1.1/.2 *is highly asymmetrical. In both of these cases there is relatively little overlap between the complement of CNEs associated with each co-ortholog pair. It is possible, therefore, that the loss of some CNEs in one co-ortholog may have consequences for further loss of elements in that gene. CRMs may not all be functionally autonomous and may interact together to actuate their regulatory role [61]. The degeneration of one or more integral CRMs from a co-ortholog could accelerate further degeneration of other CRMs that are functionally dependant on them. Under this scenario, a gene duplicate may undergo substantial loss of elements, possibly influencing further asymmetrical loss.

In addition to CNEs that have undergone full partitioning between co-orthologs, some pairs have also retained a number of overlapping CNEs that have been preserved to some extent in both copies. For co-orthologs such as *pax2.1/pax2.2*, this type of CNE constitutes the majority of CNEs located around these genes, and is a common feature in most of the other co-ortholog pairs. Overlapping CNEs appear, in general, to be longer than distinct CNEs, although at this stage the relationship (if any) between element size/conservation and its functional importance/regulatory complexity is still unknown. While some of these elements have remained virtually identical in length since duplication, others have undergone major changes both at the edges and at the core of one of the elements, suggesting information loss in one or both copies. A significant proportion of overlapping CNEs have also undergone asymmetrical rates of substitution, suggesting one copy retained the ancestral function while the other was free to evolve, possibly to a novel function.

What explanations could account for this high level of CNE retention observed between co-orthologs? The first is the possibility that some CNEs have undergone quantitative subfunctionalization. Here, degenerative mutations in both CRMs lead to a partial loss of subfunction in each element (such as a reduction in the level of expression in a specific tissue) rather than complete loss. Therefore, both elements must be maintained in the genome once the summed activity for a particular subfunction in both copies has been reduced to the original level of the ancestral gene [7,60]. Alternatively, some of these elements may function as silencers or insulators or play roles in chromatin remodeling, ensuring correct regulatory compartmentalization and control of both gene duplicates. Another explanation could be the possible inter-relations of *cis*-regulatory modules. As previously mentioned, there is a possibility that some CRMs are interrelated and act in concert to perform their function. It is possible, therefore, that the loss of a CRM critical for the function of other CRMs could lead to large-scale loss in one gene copy. For example, the partitioning of two CRMs that are functionally independent but both dependent on another CRM for correct function would lead to the retention of that critical CRM in both gene copies. Finally, it is possible that although both elements have retained general sequence identity, small nucleotide changes between the elements (such as those seen in asymmetrically evolving CNEs) may have substantial consequences for element function. Indeed in a recent pioneering study, Tümpel *et al*. [62] pinpointed subtle sequence changes within well-defined enhancers responsible for divergent expression of *hoxa2 *co-orthologs (*hoxa2*(*a*) and *hoxa2*(*b*)) in *Fugu*. Sequences of the enhancers responsible for full expression of *HOXA2 *in mice and chicken within the hindbrain were found to be generally conserved in both *Fugu *copies. Nevertheless, it was demonstrated that a small number of base-pair differences between *hoxa2*(*a*) and *hoxa2*(*b*) enhancers within several known transcription factor binding sites was sufficient to erase enhancer activity and was shown to be responsible for the lack of expression of *hoxa2*(*a*) within certain expression domains. However, the authors could not explain why the non-functional enhancers in *hoxa2*(*a*) had remained partially conserved, although they postulated they may have regulatory roles in expression domains not covered by the survey. This study highlights the power of correlating known expression differences between co-orthologs with comparative sequence analysis, especially with previous knowledge of the binding sites involved. It also highlights, as functional assays on more ancient duplicated CNEs have demonstrated [29], that sequence similarity may not always extend to functional similarity. Indeed, it is equally plausible that some of the sequence evolution seen between some overlapping CNEs is indicative of neofunctionalization. It would be of great interest for future studies to correlate any novel expression of teleost co-orthologs compared to other vertebrate homologs with changes in these elements. Finally, CNEs may represent several independent or overlapping CRMs and the loss of sequences within the CNE may be due to loss of just one of the CRMs, constituting a form of qualitative subfunctionalization

The pattern of CNE retention and evolution in these *Fugu *co-orthologs is certainly consistent with both mechanisms of subfunctionalization inherent in the DDC model. However, the extent of the contribution of each mechanism to subfunctionalization is different for each gene pair. This could be a consequence of each co-ortholog pair having followed a different evolutionary path after duplication; each under a number of different selective pressures depending on their expression and/or function as well as the influence of stochastic evolutionary events following a relaxation of evolutionary constraint due to genetic redundancy. It is clear, therefore, that confirmation of regulatory subfunctionalization in these gene pairs will require both the characterization of expression patterns for both co-orthologs (through approaches such as *in situ *hybridization) and confirmation of the regulatory potential of their surrounding CNEs through rapid *in vivo *reporter-gene assays. Currently, due to the limitations of *Fugu *as an experimental model, none of the expression profiles for the genes in this study have been characterized, which could be used to assess the extent and type of regulatory change these gene duplicates have undergone. In the more commonly used zebrafish experimental model organism gene expression profiles of two gene-pairs from this study, *pax2 *and *sox1*, have been characterized. Expression patterns of *PAX2 *co-orthologs of *PAX2 *(*Pax2a *and *Pax2b*) [63] are highly similar, although absence of *Pax2b *expression in the developing kidney as well as differences in temporal expression confirms they have undergone a level of regulatory differentiation. This appears to corroborate the pattern of element retention/partitioning seen in *Fugu pax2 *co-orthologs, where the majority of CNEs are largely conserved in both copies with a smaller number of elements partitioned between each gene. This suggests that, similar to their zebrafish homologs, they may have largely overlapping expression domains and have undergone a more subtle form of quantitative subfunctionalization through changes in their temporal expression. A recent survey of expression of the SOX B family of genes identified a level of regulatory differentiation between the zebrafish *sox1a *and *sox1b *co-orthologs [64]. The main differences in expression are temporal (for example, *sox1a *expressed in the lens a number of hours before *sox1b*), although there are also spatial differences with *sox1a *expression initiated in hindbrain and forebrain whereas *sox1b *initiates only in the forebrain. The overall expression patterns of these co-orthologs correspond closely to *SOX1 *expression in other vertebrates, indicating that changes in their expression are due to subfunctionalization rather than neofunctionalization. Our study reveals that at least half of all CNEs identified around *sox1 *co-orthologs have been partitioned, indicative of a level of subfunctionalization, while only one of the overlapping CNEs has undergone a significant level of substitution; a possible reflection on the lack of neofunctionalization in these genes. As patterns of subfunctionalization are known to occur differently between fish species, it remains for further studies of the *pax2 *and *sox1 *co-orthologs in *Fugu *to discover whether expression differences are similar to those observed in zebrafish.

The majority of regions in this study, in addition to containing CNEs derived from a teleost-specific duplication have counterparts elsewhere in the genome that derive from more ancient vertebrate-specific duplications, reflecting the complex duplication histories of their associated genes. The fact that most of these sequences are retained not only between co-orthologs (for example, *bcl11a.1 and bcl11a.2*) but also between out-paralogs (for example, *BCL11A *and *BCL11B*) spanning over half a billion years of evolution is an indicator of the potentially critical nature of these sequences to the regulation of these genes. In addition, this dataset provides many candidates for further functional studies on the evolution of these sequences and the implications of these changes on their neofunctionalized paralogs and subfunctionalized co-ortholog targets.

The role the teleost-specific genome duplication has played in the evolution of this lineage remains unclear. It is now generally accepted that the genome duplication event(s) that occurred at the origin of vertebrates played a major role in species diversity and, in particular, the huge increase in vertebrate morphological complexity [65,66]. In contrast, the more recent teleost specific genome duplication does not appear to have had the same effect, with arguably less complexity in the teleost anterior-posterior axis than in tetrapods [5]. Speciation in teleosts though is unmatched among descendants of other vertebrate lineages, with over 22,000 known species, making up over half of all extant vertebrates species [67]. This has led to suggestions that the genome duplication event may be directly responsible [14,68]. Indeed, evidence presented in a review by Taylor *et al*. [13] indicates that ployploidized members of the Salmon family and Catostomidae (sucker fish) exhibit higher degrees of speciation than members of the same family that remain diploid. Subfunctionalization has been proposed as a likely mechanism for this increased rate of speciation since differential resolution of subfunctions in multiple gene pairs would lead to reproductive incompatibility due to a reduction in hybrid fitness [69]. Evidence for such lineage-specific subfunction partitioning has been demonstrated for a small number of genes (for example, divergent expression of *sox9 *in stickleback compared to zebrafish [18]), but large-scale studies will be required to resolve the degree of subfunctionalization that took place before and after divergence within the teleost lineage. Furthermore, if lineage speciation is driven by differential subfunctionalization, we might expect the pattern of CRM evolution and partition/retention for the *Fugu *genes discussed here to be different to those in other fish species. The recent release of a number of divergent draft teleost genomes, including those of zebrafish, medaka and stickleback, should allow further studies in this direction. Furthermore, the approach and analysis used in this study can be extended for use in any situation where genomic regions surrounding duplicated genes can be compared to an orthologous region that has remained single copy. This may be particularly useful for inter-teleost comparisons, where co-ortholog genes have been differentially retained since the whole-genome duplication prior to the teleost radiation.

## Conclusion

Regulatory subfunctionalization is considered to be a major mechanism for the retention of gene duplicates in the genome. This work provides the first large-scale identification and analysis of putative *cis*-regulatory elements through comparative genomics between duplicated genes using the *Fugu *genome as a model. Using seven pairs of fish-specific gene duplicates we showed that all pairs have undergone a level of element partition consistent with one of the main mechanisms proposed for regulatory subfunctionalization. In addition, the regulatory elements in this study may have undergone more subtle levels of subfunctionalization through differential loss of element content and asymmetrical rates of substitution. In addition to presenting this work as an analysis in its own right, the methods in this study can be extended to any similar study in which regions derived from an intra-genomic duplication can be compared to one or more related genomes in which the orthologous region has remained single-copy.

## Materials and methods

### Identification of CNE-containing co-orthologous regions in the *Fugu *genome

An initial set of 2,330 CNEs with little or no evidence of transcription or RNA secondary structure were identified using a whole-genome comparison of the *Fugu *(assembly v4) and human genomes (assembly v.36) as described in [29]. CNEs in the human genome were grouped into clusters so that each CNE was no more than 400 kb in distance from another CNE in the cluster. Clusters of CNEs in the human genome made up of hits from two non-contiguous *Fugu *scaffolds (that is, two separate locations in the *Fugu *genome) were considered further. Previously, we reported that the genes found closest to CNEs are statistically over-represented for Gene Ontology (GO) annotations [70] relating to transcriptional regulation and/or development (*trans-dev*) [26]. Genes within each of these clusters in the human genome (including the closest gene either side of the cluster) were considered to be *trans-dev *if they contained any of these over-represented GO annotations. To avoid ambiguities in associating CNEs to genes, we selected only those regions containing a single *trans-dev *gene within the CNE cluster. Ten pairs of *Fugu *scaffolds conformed to these criteria. Seven regions containing the largest number of CNEs in addition to well defined orthologous sequence within each *Fugu *region (that is, those that contain genes neighboring the CNE cluster) were selected for further analysis. These scaffolds contain CNEs that are conserved in the vicinity of the following *trans-dev *genes in the human genome: *BCL11A*, *EBF1*, *FIGN*, *PAX2*, *SOX1*, *UNC4.1 *and *ZNF503*. *Fugu *protein sequences from the corresponding orthologs were obtained from Ensembl Compara (v36), except in the case of *PAX2 *where only partial sequences were present. In these cases, tBLASTn searches using known protein sequences from zebrafish *pax2 *co-orthologs *pax2.1 *(SPTR: PAX2_BRARE) and *pax2.2 *(SPTR: O93370) were used to identify the *Fugu *protein sequence.

To verify that these genes were phylogenetically co-orthologous to mammalian copies we carried out multiple alignments of each pair of *Fugu *proteins together with available orthologs from human, mouse, rat, dog and chicken using CLUSTALW (v1.83) [71] downloaded from Ensembl Compara (v36) unless otherwise stated. In addition we used all available orthologs from zebrafish. Two of these are previously experimentally characterized co-orthologs and were downloaded from the SwissProt protein database (*pax2.1*, PAX2_BRARE; *pax2.2*, O93370; *sox1a*, Q4V997; *sox1b*, Q2Z1R2). The remaining novel zebrafish orthologs were downloaded using Ensembl Compara. In the cases of *BCL11A*, *FIGN *and *ZNF503 *only a single ortholog was identified by Ensembl. In the case of *EBF1*, no zebrafish ortholog could be identified, and was, therefore, replaced by a single ortholog from the stickleback genome. The closest available invertebrate ortholog of each gene in either *Ciona *(ci), *Drosophila *(dm) or *Caenorhabditis elegans *(ce) was used as an outgroup (*BCL11A*, *LD11946p *(dm); *EBF1*, coe (ci); *FIGN*, *CBG21866 *(ce); *PAX2*, *pax258 *(ci); *SOX1*, *soxNRA *(dm); *UNC4.1*, *unc4 *(ci); and *ZNF503*, *noc *(dm)).

The closest paralog from human, mouse, rat, dog, chicken and *Fugu *in each case was also included as an outgroup in each alignment (that is, *BCL11A:BCL11B*, *FIGN:FIGN1L*, *PAX2:PAX5*, *SOX1:SOX3*, *EBF1:EBF3*, *ZNF503:ZNF703*). The closest related paralog was defined as the highest significantly scoring non-ortholog high scoring pair (HSP) in a BLASTp search of the human protein sequence against the SwissProt/trEMBL nr protein database. No closely related paralog could be identified for *UNC4.1*. A phylogenetic tree was created from each alignment using the neighbor joining (NJ) method and 1,000 bootstrap replicates using MEGA v3.1 [72].

### *Fugu *co-ortholog gene nomenclature

*F. rubripes *is not a good experimental model organism due to difficulties in captive breeding and experimental manipulation. Consequently, few of its genes have been experimentally validated. Most gene predictions in the *Fugu *genome therefore remain novel, uncharacterized and have no current gene name. For the purposes of this study we decided upon a naming scheme for the *Fugu *co-orthologs that uses the same name as the human ortholog (for example, *SOX1 *= *sox1*) together with a number that denotes the specific co-ortholog (for example, *sox1.1/sox1.2*). This naming convention is similar to that used in early studies of zebrafish co-orthologs (for example, *pax2.1 *[63]) but which has now been superseded by a naming convention using letters (for example, *pax2a*) (ZFIN gene nomenclature guidelines [73]). We therefore used a number-based nomenclature to distinguish *Fugu *co-orthologs from zebrafish co-orthologs. For those genes in which zebrafish co-orthologs had previously been characterized (*pax2a/pax2b*, *sox1a/sox1b*) we named *Fugu *equivalents by their phylogenetic similarity to these characterized zebrafish genes as ascertained through phylogeny. So, as an example, *PAX2 *co-orthologs were identified on *Fugu *scaffolds 86 and 59 (assembly v4; Figure 1d). The phylogeny identified the protein encoded on S86 as closest to zebrafish *pax2a *and that encoded on S59 as closest to *pax2b *(Figure 1c); therefore, the gene on S86 was named *pax2.1 *and the gene on S59 was named *pax2.2*. The rest of the co-orthologous sets that did not have characterized zebrafish equivalents were assigned names randomly. It is important to note this nomenclature is used purely to distinguish the genes and has no functional significance.

### Identification of CNEs in *Fugu *co-orthologous regions

CNE clusters derived from the whole-genome alignment were used to define the extent of sequence in both human and *Fugu *for use in more sensitive multiple alignments. Regions up to the next known gene from the most peripheral CNEs in each cluster were extracted in both human and *Fugu *using the Ensembl API [74]. Special attention was paid to include the same orthologous region between co-orthologous pairs to ensure equivalent comparison. In situations where the full extent of the region could not be identified in one of the co-orthologs due to the location of the region at the end of a scaffold (for example, scaffold_86, *znf503.1*; Additional data file 1), only CNEs identified up to the same orthologous region (estimated by the presence of nearby genes) in the second co-ortholog were used for comparative analyses. Orthologous sequences corresponding to each human region were similarly extracted in mouse (assembly v34) and rat (assembly v3.4). All genomic sequences were orientated prior to alignment so that the *trans-dev *gene was in positive orientation and masked for known repeats and low complexity regions using RepeatMasker and the relevant species-specific repeat library. Multiple alignments for the discovery of conserved sub-sequences located in the same relative order and orientation were carried out using the MLAGAN alignment toolkit [75] with translated anchoring and the phylogenetic guide tree '((human (mouse rat)) fugu)'. Pairwise glocal alignments to uncover conserved elements that may have undergone rearrangements (and are, therefore, no longer in the same relative order along the sequence) or inversions between *Fugu *and all other organisms utilised in the MLAGAN alignment were carried out in a pairwise fashion using Shuffle-LAGAN [76] with default parameters. Each pair of *Fugu *co-orthologous regions was aligned to the same orthologous mammalian sequence. CNEs were identified from the alignments using the VISTA program [77] as regions with at least 65% identity over 40 bp using *Fugu *as the baseline sequence. CNEs were filtered further to include only those that were conserved in human and at least one rodent.

### Identification of overlapping and distinct CNEs between *Fugu *co-orthologous regions

The human sequence was used as a reference in order to ascertain whether CNEs identified from each co-ortholog region overlapped the same human sequence (termed 'overlapping') or were conserved in only one co-ortholog (termed 'distinct'). CNEs between co-orthologs were considered overlapping if the conserved sequence overlapped the same position in the human genome by at least 20 bp. *Fugu *CNEs that were defined as 'distinct' to one co-ortholog were used as query sequences against the alternative *Fugu *co-orthologous genomic region using the CHAOS local aligner [75] on both strands with the following parameters: word length 10, score cut-off 10, degeneracy tolerance 1, rescoring cut-off 1,000, and BLAST-like extension on. Resulting alignments were filtered to retain only those with at least 65% identity over 40 bp.

### Evolution of overlapping CNEs

#### Element length

To ensure equivalent comparison, the length of the human CNE was used when measuring changes in element length between CNEs conserved in both co-orthologs. For each pair of overlapping CNEs with a one-to-one relationship (that is, each CNE overlapped one other CNE), the proportion (*P*) of the length of the overlap compared to the full length of each CNE was calculated using:

*P *= *ov/len*

where *ov *is the length of the overlap between co-orthologous CNE (in bp) and *len *is the full length of the CNE. Values of *P *that tend towards 1 indicate all or the majority of the element is contained within the overlap while those tending towards 0 indicate only a small proportion of the element is contained within the overlapping region.

#### Sequence evolution

To compare the evolutionary rates of *Fugu *co-orthologous copies against the single human copy (representing the ancestral sequence) we used all 'overlapping' co-orthologous CNEs. For those CNEs that did not have a one-to-one relationship (for example, in cases where two or more CNEs in one region overlapped a single CNE in another) we treated each individual overlap region independently. Multiple alignments were created for each co-ortholog CNE individually together with orthologous sequence from human, mouse, rat, dog and chicken (where available) to produce the best mapping of orthologous bases. The human sequence from the overlap detected was used to extract corresponding sequence within each multiple alignment for each co-orthologous *Fugu *copy together with those of orthologous sequences from the other vertebrates. These sequences were then realigned together using DIALIGN (v2.2) [78] and all gapped positions removed using the Gblocks program (v0.91b) [79]. A Tajima relative rate test of each pair of *Fugu *co-orthologous copies against the single human sequence was carried out as described in [57]. Only sequences that showed at least 4 independent changes in one of the elements and a *p *value ≤ 0.05 were considered to have undergone significant change.

#### Identification of CNEs duplicated at the origin of vertebrates

All human CNE sequences were searched against the human genome using BLAST with sensitive parameters (word size 8, mismatch penalty -1) to identify CNEs that have more than a single match (e-value ≤ 1 × 10^-4^) in the human genome. Paralogs were identified within 1.5 Mb of each hit using the method set out in [29]. The probability of the enrichment for overlapping CNEs within the dCNE set was calculated using a χ^2 ^test with expected numbers for each type of CNE (overlapping versus distinct) calculated from the proportion of each within the whole CNE dataset (381:430 = 0.469:0.531).

## Additional data files

The following additional data are available with the online version of this paper. Additional data file 1 is a comparison of the CNEs and genic environment between *Fugu *co-orthologs of *znf503.1 *and *znf503.2*. Additional data file 2 is a bar chart showing that changes in the number of CNEs between co-orthologs correlates with changes in the size of the genomic region in which they are identified. Additional data file 3 is a full table of results of the relative rate tests for all overlapping co-orthologous CNEs.

## Supplementary Material

Additional data file 1 Comparison of the CNEs and genic environment between *Fugu *co-orthologs of *znf503.1 *and *znf503.2* Click here for file

Additional data file 2 Changes in the number of CNEs between co-orthologs correlates with changes in the size of the genomic region in which they are identified Click here for file

Additional data file 3 Results of the relative rate tests for all overlapping co-orthologous CNEs Click here for file

## Figures and Tables

**Table 1:** Co-ortholog nomenclature and genomic locations in the *Fugu genome*

Human gene*	Co-ortholog name^†^	*Fugu *scaffold (S) location (kb)^‡^	Length (kb)^§^	Prop 'N's (%)^¶^	*Fugu *protein accession code^¥^
*BCL11A*	*bcl11a.1*	**S113**: 140.8-518.9	378.1	2.98	NEWSINFRUP00000142044
	*bcl11a.2*	**S62**: 603.7-740.4	136.7	0.18	NEWSINFRUP00000144873
*EBF1*	*ebf1.1*	**S97**: 400.4-483.3	82.9	0.82	NEWSINFRUP00000127762
	*ebf1.2*	**S71**: 999.3-1,091.7	92.4	1.90	NEWSINFRUP00000148373
*FIGN*	*fign.1*	**S36**: 382.6-486.8	104.2	0.16	NEWSINFRUP00000153680
	*fign.2*	**S46**: 126.9-219.9	93	0.39	NEWSINFRUP00000177971
*PAX2*	*pax2.1*	**S86**: 541.7-669.8	128.1	0.29	-
	*pax2.2*	**S59**: 768.9-898.3	132.7	3.59	-
*SOX1*	*sox1.1*	**S42**: 1,020-1,105	85	1.49	[Swiss-Prot: Q6WNU3_FUGRU]
	*sox1.2*	**S313**: 107.2-174.9	67.7	8.9	[Swiss-Prot: Q6WNU2_FUGRU]
*UNC4.1*	*unc4.1.1*	**S15**: 761.1-825.5	61	0.32	NEWSINFRUP00000154395
	*unc4.1.2*	**S40**: 1,435-1,537	102	0.96	NEWSINFRUG00000161008
*ZNF503*	*znf503.1*	**S86**: 7-220	213	3.64	NEWSINFRUP00000181530
	*znf503.2*	**S59**, **S29 **(all)	148.5	3.22	NEWSINFRUP00000181454

*Name of human gene ortholog. ^†^Nomenclature of novel *Fugu *co-orthologs. ^‡^Location and extent of *Fugu *genomic scaffold used in multiple alignment. ^§^Length of *Fugu *genomic region used in multiple alignment. ^¶^Proportion of *Fugu *genomic region that is made up of unfinished sequence (that is, runs of 'N's). ^¥^The protein accession code for each co-ortholog. These were derived either from Ensembl (v40.4b) or from SwissProt. Protein sequences for *pax2.1 *and *pax2.2 *were incomplete in both Ensembl and SwissProt and were reconstructed using alignments of full-length amino acid sequences from other species.

**Table 2:** Tajima relative rate tests of overlapping co-orthologous CNE in *Fugu*

Gene region*	No. of overlapping pairs^†^	No. of CNE pairs with *p *> 0.05^‡^	No. of CNE pairs with *p *≤ 0.05^§^		% of CNE pairs with *p *< 0.05^¶^
				
			Co-ortholog 1	Co-ortholog 2	
*BCL11A*	20	15	1	4	25
*EBF1*	7	5	1	1	21
*FIGN*	28	21	5	2	25
*PAX2*	43	34	4	5	21
*SOX1*	6	5	0	1	17
*UNC4.1*	20	15	0	5	25
*ZNF503*	84	62	5	17	26

*Gene region. ^†^Total number of overlapping CNEs within gene region. ^‡^Numbers of overlapping CNE pairs with no significant difference in substitution rates (that is, *p *values of > 0.05). ^§^The number of overlapping CNEs that exhibit a significant difference in substitution rate (that is, *p *value ≤ 0.05) in the CNE sequence in the vicinity of one co-ortholog over that in the other. ^¶^The percentage of overlapping CNEs with significantly different substitution rates in either co-ortholog as a proportion of the total number of overlapping CNEs.

**Table 3:** Co-ortholog CNEs that are also conserved in the vicinity of *trans-dev *paralogs in the human genome

Gene region	Co-ortholog 1	Co-ortholog 2	Gene paralog in the vicinity of the dCNE(s)
*BCL11A*	CRCNE00002445	CRCNE00004614	*BCLL1B*
	CRCNE00002557	-	
	CRCNE00002548	CRCNE00004648	
	CRCNE00002544	CRCNE0004643	
		CRCNE0004644	
	CRCNE00002540	-	
*EBF1*	CRCNE00010771	-	*EFB3*
	-	CRCNE00010818	
	CRCNE00000027	CRCNE00010823	
	CRCNE00010778	CRCNE00010827	
	CRCNE00010787	-	
*EBF1*	CRCNE00010772	CRCNE00010820	*EBF1/2/3/4*
*PAX2*	CRCNE00000064	CRCNE00000133	*PAX8*
*PAX2*	CRCNE00000071	CRCNE00000147	*PAX5*
	CRCNE00000090	CRCNE00000165	
	CRCNE00000092	CRCNE00000167	
	CRCNE00000099	CRCNE00000174	
*SOX1*	-	CRCNE00001926	*SOX2*
*ZNF503*	CRCNE00010112	CRCNE00004977	*ZNF703*
	CRCNE00010147	CRCNE00004994	
	CRCNE00010126	CRCNE00005024	
	CRCNE00010167	-	
	CRCNE00010170	-	
	CRCNE00010187	-	
	CRCNE00010180	-	
	CRCNE00010176	CRCNE00005013	
		CRCNE00005015	
	CRCNE00010165	CRCNE00005011	
	CRCNE00010161	CRCNE00005008	
	CRCNE00010046	CRCNE00004906	
	CRCNE00010156	CRCNE00005003	
	CRCNE00010120	CRCNE00004986	

CNEs in each co-ortholog are referred to by their CONDOR database identifiers [53]. Each CNE was considered duplicated if the human sequence they are conserved to shows significant hit to a sequence elsewhere in the genome through BLAST. Any gene in the vicinity (<1.5 Mb away) of the BLAST hit that is paralogous to genes within a window of the same size around the query CNE is shown in the final column.
