# Supplementary material for: Comparative genomics using Fugu reveals insights into regulatory subfunctionalization
Source: Genome Biol. 2007 Apr 11;8(4):R53. doi: 10.1186/gb-2007-8-4-r53 (PMC1896008; doi:10.1186/gb-2007-8-4-r53)
Supplement: Additional data file 1 — Comparison of the CNEs and genic environment between Fugu co-orthologs of znf503.1 and znf503.2 [file gb-2007-8-4-r53-S1.doc]

**Additional file 1. CNEs and genic environment around *Fugu* co-orthologs *znf503.1* (top) and *znf503.2* (bottom)**. Sequence length is shown across the top track in kilobases. Gene structures are shown on the CDS (or coding sequence) track and positions of all CNEs in *Fugu* are shown in blue boxes. CNEs located close together are ‘bumped’ onto lower lines. For *znf503.1*, it was only possible to retrieve sequence as far as the region after *vdac2*. The corresponding region in *znf503.2* is shown within the red dashed-line box. A number of CNEs in *znf503.2* were identified beyond this but these were not included in the comparative analyses. The region around *znf503.2* has undergone a level of CNE loss (with ~16% more CNEs identified around *znf503.1* compared to around *znf503.2* (within the red box)) together with reduction of genomic space (~300Kb in *znf503.1* compared to ~145Kb in *znf503.2*). The *znf503.2* region has also undergone the loss of the *c10orf11* ortholog. Only a small match to the mRNA sequence of human *C10orf11* remains suggesting the gene in this copy underwent non-functionalization and was lost from the genome.
