# Supplementary material for: Comparative genomics using Fugu reveals insights into regulatory subfunctionalization
Source: Genome Biol. 2007 Apr 11;8(4):R53. doi: 10.1186/gb-2007-8-4-r53 (PMC1896008; doi:10.1186/gb-2007-8-4-r53)
Supplement: Additional data file 2 — Changes in the number of CNEs between co-orthologs correlates with changes in the size of the genomic region in which they are identified [file gb-2007-8-4-r53-S2.doc]

**Additional file 2. Changes in the number of CNEs conserved in each *Fugu* co-ortholog correlates with changes in the size of genomic DNA in which CNE are identified.** Description: Genomic distance is measured as the length of DNA between the furthest overlapping CNEs (i.e. CNEs conserved in both co-orthologs) upstream and downstream of the co-ortholog gene in kilobases. The number of CNEs represents only those CNEs that were located within this defined genomic region.
